# Exploring intra-varietal variation for complex traits in grapevine (*Vitis vinifera* L.)

**DOI:** 10.1007/s00122-025-05088-3

**Published:** 2025-11-14

**Authors:** Hannah Robinson, Timo Strack, Maximilian Schmidt, Paolo Callipo, Mariem Nsibi, Joachim Schmid, Ernst Rühl, Hans-Peter Piepho, Kai P. Voss-Fels

**Affiliations:** 1 Department of Plant Breeding, Hochschule Geisenheim University, Geisenheim, Germany; 2 Biostatistics Unit, Institute of Crop Science, University of Hohenheim, Stuttgart, Germany

## Abstract

**Key message:**

Centuries of clonal propagation have shaped remarkable intra-varietal genetic diversity in grapevine, offering valuable opportunities to dissect complex traits and accelerate genetic improvement while safeguarding varietal integrity.

**Abstract:**

Climate change poses significant challenges to global grapevine (*Vitis vinifera* L.) production, highlighting the urgent need for adaptive breeding strategies to accelerate genetic improvement. While clonal propagation preserves varietal identity and heterozygosity, it also limits the rate of genetic gain due to prolonged breeding cycles. This study assessed phenotypic and genetic variation within eight clonal populations of key grapevine varieties (Pinot Blanc, Pinot Gris, Pinot Noir, Pinot Noir Précoce, Riesling, Müller-Thurgau, Auxerrois, and Savagnin Rose) using 14 years of data collected in Germany across six agronomic, quality, and disease-related traits. Estimates of broad-sense heritability, genetic correlations, and key variance components were derived using linear mixed models. Substantial intra-varietal phenotypic variation was observed across all traits, with moderate to high heritability estimates, confirming that a meaningful proportion of the phenotypic variation can be attributed to the genetic differences among clones. Substantial year and year-by-field variance and interaction components were found to contribute to the total phenotypic variance for most traits, aligning with previous reports of substantial genotype-by-environment interaction in clonal grapevine populations. Genetic correlations revealed both strong positive and strong negative trait relationships, emphasising the importance of informed multi-trait selection strategies. The results highlight considerable potential to enhance clonal selection by integrating predictive breeding tools such as genomic and phenomic selection. Optimisation-based multi-trait selection approaches also offer promising alternatives to traditional index methods, particularly in the context of negative trait correlations. Ultimately, the high intra-varietal genetic variation uncovered in this study represents a valuable resource for improving adaptation to future environments while maintaining varietal integrity in grapevine.

**Supplementary Information:**

The online version contains supplementary material available at 10.1007/s00122-025-05088-3.

## Introduction

Grapevine (*Vitis vinifera* L.) stands apart in agriculture due to the great importance of traditional cultivars, like Pinot Noir and White Riesling, representing living genetic heritage propagated continuously for centuries. In contrast to many agricultural sectors that readily adopt newly bred varieties, the global wine industry is uniquely anchored to these ancient cultivars. Market identity, regional typicity, and consumer recognition are inextricably linked to these traditional varieties, creating significant barriers to the adoption of new cross-bred cultivars (Töfper and Trapp 2022).

Despite this, the need for genetic improvement persists and is driven largely by the urgent challenges posed by climate change. Rising temperatures, in particular, are accelerating phenology and increasing vulnerability to spring frosts, while also increasing the speed of sugar accumulation in grapes, resulting in high alcohol content and altered flavour profiles in wines (Van Leeuwen et al. 2024; Baltazar et al. 2024). In addition, altered precipitation patterns and increased humidity favour the spread of fungal diseases such as downy mildew (*Plasmopara viticola*), powdery mildew (*Erysiphe necator*), and botrytis bunch rot (*Botrytis cinerea*), leading to significant impacts on yield and fruit quality (Van Leeuwen et al. 2024). Without targeted intervention it is very apparent that global grapevine production is on an unsustainable trajectory.

Faced with the limited market acceptance of new varieties, grapevine breeders have pivoted towards clonal selection as a key avenue to identify and harness complex trait variation for the genetic improvement of traditional cultivars (Schmidt et al. 2025). This approach contrasts sharply with clonal selection programs in many other clonally propagated species (e.g. potato, strawberry), where the objective is typically to generate new varieties through hybridisation, followed by selection and multiplication of the best resulting individual clone. In grapevine, however, clonal selection focuses on identifying, evaluating, and propagating superior clones that have emerged *within* the existing populations of ancient cultivars (Callipo et al. 2025). This intra-varietal diversity originates from the gradual accumulation of somatic mutations and epigenetic modifications over centuries of vegetative propagation (Douhovnikoff and Dodd 2015; Vondras et al. 2019), leading to phenotypic differences among clones classified under the same variety name (Pelsy 2010). Somatic mutation is a genetic alteration occurring in non-reproductive cells, frequently accumulating over time through repeated cycles of clonal propagation and cell division. While epigenetic modifications such as DNA methylation and histone modification can induce phenotypic variation among genetically identical clones without altering the DNA sequence, typically arising in response to environmental stresses and potentially being heritable through vegetative propagation (Berger et al. 2023). Practices like polyclonal selection further leverage this existing diversity by propagation of a group of genetically distinct but complementary clones within a variety to enhance vineyard resilience while maintaining varietal identity (Martins and Gonçalves 2015).

Traditional clonal selection is exceedingly slow, where the average grapevine breeding cycle can span up to, or even exceed, 25 years, severely limiting the rate of genetic gain. This is largely due to the use of clonal propagation to preserve high heterozygosity, coupled with a prolonged juvenile phase of up to five years, which delays early-stage phenotypic selection for key yield and quality traits. In phylloxera-prone regions, the need for grafted vines can prolong this process by one to two years. In addition, the perennial nature of grapevine makes field evaluations more resource-intensive, while prolonged exposure to varying environmental conditions underscores the importance of extensive multi-year assessments. These constraints are further compounded by the inherent inefficiencies of clonal propagation, requiring labour-intensive cutting, grafting, phytosanitary testing and nursery management, and when combined with limited resources typical of public breeding programs, restrict the scalability and pace of multi-environment field evaluations.

Prior research consistently demonstrates substantial phenotypic variation within clonal populations of diverse grapevine varieties, highlighting potential for clonal selection. Studies investigating cultivars like Cabernet Franc (Van Leeuwen et al. 2012), Grenache (Buesa et al. 2021), Malbec (van Houten et al. 2020), and Tempranillo (Arrizabalaga et al. 2018; Portu et al. 2024) report significant intra-varietal diversity for yield and quality traits, often even within relatively modest population sizes (e.g. 9–33 clones). This finding is echoed in large-scale surveys across multiple French regions (Neethling et al. 2023) and among numerous ancient Portuguese varieties, where considerable diversity, particularly for yield (e.g. genotypic coefficient of variation up to 59%), was evident (Gonçalves and Martins 2022). However, investigations into phenotypic correlations remain limited with the except of one study in Tempranillo where a negative yield-malic acid relationship was observed alongside expected positive correlations among acidity component traits (Portu et al. 2024). Crucially, despite documenting phenotypic variation, the majority of previous work has generally not partitioned this variance to quantify the specific contribution of the clonal genetic effect. Addressing this gap, a study by Laidig et al. (2009) analysed 30 White Riesling clones across a large scale, encompassing 16 locations over 36 years. This research is significant for explicitly partitioning variance using mixed models on extensive, unbalanced multi-environment trial data, thereby estimating the specific contribution of the clonal genetic effect. Their analysis quantified this clonal effect, finding it accounted for a small proportion (less than 1%) of the total phenotypic variation for key traits like yield, sugar content (total soluble solids), and acidity, with environmental factors (location, year, and their interactions) explaining the vast majority (around 95%). Accurately partitioning this variance is thus a critical first step towards leveraging clonal diversity through selection technologies.

Integrating modern predictive breeding approaches, such as genomic selection (GS) with the rich genetic resources found in varietal clonal populations presents a powerful strategy for accelerating genetic gain specifically tailored to the grapevine industry’s constraints. For example, GS can potentially shorten the evaluation cycle by predicting clonal genetic merit early using genome-wide markers (Meuwissen et al. 2001). However, the success of such predictive models rests on accurately characterising the genetic architecture of traits within large, accurately phenotyped training populations. Addressing the limitations of previous research, namely the need for larger population sizes, multi-year trait evaluation, and robust variance component analysis, is crucial for building effective predictive models for clonal selection in grapevine.

This study aims to quantify the extent of phenotypic variation within eight commercially important varietal clonal populations by assessing multi-year trait data for yield, sugar, and main organic acids, as well as disease susceptibility. Using a linear mixed model framework, the study estimates the contribution of genetic effects to trait variation and evaluates broad-sense heritability across traits and populations. Additionally, trait genetic correlations are examined to identify key relationships influencing selection strategies, providing insights into opportunities for optimising clonal selection and accelerating genetic improvement in grapevine.

## Materials and methods

### Collection and establishment of clonal populations

Eight clonal populations evaluated in this study represent the varieties Auxerrois (*n* = 87), Müller-Thurgau (*n* = 123), Pinot Blanc (*n* = 40), Pinot Gris (*n* = 161), Pinot Noir (*n* = 227). Pinot Noir Précoce (Pinot Précoce; *n* = 107), Riesling (*n* = 938), and Savagnin Rose (Savagnin; *n* = 98). Individual clones within each group were selected from historic vineyards across Germany, France, Austria, and Luxembourg (Supplementary Fig. 1), based on advantageous agronomic traits. All clonal populations were screened for major grapevine viruses using standard ELISA and PCR-based diagnostics. The panel included Grapevine Pinot gris virus (GPGV), Rupestris stem pitting-associated virus (RRsV-ch), Grapevine leafroll-associated virus 1 (GLRaV-1), Arabis mosaic virus (ArMV), and Grapevine fanleaf virus (GFLV). This testing ensured that infected clones were identified, and removed from the analysis to confirm robustness of results. Following virus testing and varietal confirmation, clones were propagated from single-bud cuttings and grafted onto phylloxera resistant rootstock varieties (Supplementary Tables 1 and 2). The varietal clonal populations were planted in the experimental vineyards of Geisenheim University, across eight fields where within-field replication was absent but partial replication across the field present (Supplementary Table 1, Fig. 2A), and depending on the clone and population, clones were planted across years with the year of planting recorded. All vineyard fields were managed consistently according to standardised viticultural practices, i.e. uniform canopy, pruning level, soil, and cover crop management, identical plant protection regimes, and no yield-reducing interventions, and were laid out in a two-dimensional format, with rows in the horizontal direction and plots in the vertical direction (Supplementary Fig. 2B). Each vineyard plot consisted of three vines per clone (Supplementary Fig. 2C), allowing for within-plot replication to account for small-scale heterogeneity in soil conditions, plant spacing, or microclimate.

### Phenotypic trait observations

The eight clonal populations were evaluated for yield, juice quality parameters and botrytis susceptibility, across 14 seasons from 2009 until 2023. Due to breeding program resource constraints, not all clones were sampled for all traits in all 14 years, however all but one clonal population have at least 9 years of trait data. Supplementary Fig. 2B graphically depicts the distribution of clonal populations across the Geisenheim University experimental vineyard as well as the sampling frequency of each plot across the duration of the study (Supplementary Table 1).

Six grapevine breeding priority traits were phenotyped as part of the study, including total yield (g/m^2^), total soluble solids (brix; °Brix), total acidity (g/L; titratable acidity expressed as tartaric acid), malic (g/L) and tartaric acid content (g/L) and botrytis infection (%). To measure the juice quality parameters, clusters from each plot were pressed using a 20 L hydraulic basket press (Speidel Tank- und Behälterbau GmbH, Ofterdingen, Germany). Prior to coarse filtration (16 μm Munktell 33/N, 90 g m⁻^2^ folded filter; Ahlstrom, Helsinki, Finland), 8 mL hL⁻^1^ of pectolytic enzymes (Trenolin 4000 DF; Erbslöh GmbH, Geisenheim, Germany) were added to the juice. A single bulk juice sample per plot was collected for analysis. Brix, total acidity, malic acid, and tartaric acid concentrations were measured using Fourier-transform infrared (FTIR) spectroscopy with a Winescan FT2 spectrometer (FOSS, Hillerød, Denmark) and an in-house grape must calibration. Disease severity of Botrytis bunch rot was assessed visually according to the EPPO standard PP 1/17(3) (*Botryotinia fuckeliana* on grapevine; EPPO 2004). A single overall severity score was recorded per plot; however, the identity of the annotator was not documented, and therefore annotator effects could not be included as a factor in the downstream modelling of Botrytis bunch rot severity.

### Raw data curation

The raw data collect across 14 years was compiled individually for each of the eight varietal clonal populations and herein all populations were analysed separately. All data analyses were performed using R (R Core Team 2024). To remove outliers prior to calculating descriptive statistics and graphical representation of raw observations, a base linear mixed model was fit to each population using the following equation:

1 $$y= {\rm X}\tau + {\rm Z}u+e$$

where ${\rm X}$ and ${\rm Z}$ are the design matrices associated with the fixed $\tau$ and the random $u$ effects, and $e$ is the residual error term. In this base model, the intercept was fitted as a fixed effect, while clone, year, and field effects were modelled as random. A separate residual variance was estimated for each individual year, allowing for year-specific plot error. Following base model convergence, the model was updated to estimate the scaled residuals, and plot observations that were above or below a standardised residual threshold value of 4 were identified as outliers and removed. The base model was updated and the outlier detection process repeated until no observations exceeded the scaled residuals threshold. All standard assumptions of the linear mixed model, including the normality of residuals, homogeneity of variances, and independence of errors, were assessed through diagnostic plots and deemed to be satisfactorily met. The exception was the trait botrytis, which required a logarithmic transformation (i.e. log(1 + *x*)) to reduce skewness and stabilise residual variance, thereby improving adherence to model assumptions. The base linear mixed model was fit in the R package ‘ASReml-R’ (Butler et al. 2009) and all graphical outputs created using ‘ggplot2’ (Wickham 2016).

### Univariate linear mixed model and associated variance estimates

Post-raw data curation, a final linear mixed model, following the equation outlined in (1) was fit individually for each trait within each population. In the final model, for all populations, the fixed effects included an overall intercept and the random effects included effects for clone, year, field and a diagonal variance structure fit to the year-by-field interaction. The residuals were modelled as year-by-plot with a diagonal variance structure assigning a separate variance for each year. The diagonal structure accommodates heterogeneous variances across levels of a factor (e.g. year) while assuming zero covariances. For populations Auxerrois, Müller-Thurgau and Riesling, planting year was also fitted as a continuous fixed effect, and was tested for significance at *p* < 0.05 using the Wald test statistic. For the Pinot Blanc, Pinot Gris, Pinot Noir, Pinot Précoce and Savagnin Rose populations, the planting year was not found to be significant and was not included as a fixed effect in the model. Rootstock was not modelled separately due to its confounding with field. Within each varietal population, fields were either planted with a single rootstock or had highly unbalanced rootstock distributions (Supplementary Table 1). Consequently, any rootstock effects are captured within the field term. Based on log-likelihood ratio tests and Akaike Information Criterion (AIC), the diagonal variance structure provided the best fit for both the random year-by-field interaction and the residual at the year-by-plot level. Although a correlated heterogeneous variance structure was considered for the year component of the year-by-field random effect, to account for potential heterogeneity and covariance between fields observed in different years, the model failed to converge, likely due to data sparsity in certain year–field combinations. Given the perennial nature of grapevine and the expectation of residual correlation across years within plots, a first-order autoregressive residual structure (ar1), was fit at the year-by-plot level. While a weak negative correlation was estimated, the model fit was not improved based on the REML log-likelihood and AIC. To better accommodate heterogeneity in residual variance across years, an ar1h structure was also tested; however, due to the sparsity and imbalance of the data and especially in respect to the variance in sampling the same plot each year, the model failed to converge. As such, a simpler diagonal residual structure was retained. The final model ASReml-r script is detailed in Supplementary Materials. Following model convergence, variance components and associated *z*-ratios were extracted. Multi-year empirical best linear unbiased predictors (E-BLUPs) were obtained for each clone and the scaled average pairwise prediction error variance (${A}_{tt}$). Broad-sense heritability (*H*^2^) was calculated based on these components using the method described by Cullis et al. (2006), which accounts for unbalanced data and the prediction uncertainty:

2 $${H}^{2} =1- \frac{{A}_{tt}}{{2*\sigma }_{\text{g}}^{2}}$$

where ${A}_{tt}$ refers to the mean of average variance of a difference of the E-BLUPs and ${\sigma }_{\text{g}}^{2}$ the genetic variance. The genotypic coefficient of variation (CV_G_%) was calculated as:

$${\text{CV}}_{\text{G}}\%= \frac{\sqrt{{\sigma }_{\text{g}}^{2}}}{\overline{x} } \times 100$$

where ${\sigma }_{\text{g}}^{2}$ is the estimated genetic variance component, and $\overline{x }$ is the model-based marginal mean (best linear unbiased estimate of the population means).

### Bivariate linear mixed models to estimate genetic correlations

A series of bivariate linear mixed models were fitted to estimate genetic correlations between each pairwise trait combination within each clonal population. Prior to bivariate analysis, trait values were standardised using *z*-score transformation, centring by the population mean and scaling by the standard deviation to achieve a mean of zero and a standard deviation of one. The fixed, random, and residual terms followed the univariate model structure described above. To model trait-specific variance and covariance across model components, 2 × 2 unstructured variance–covariance matrices were fitted to the following random effects: trait-by-clone, trait-by-year, and trait-by-year-by-field. For the trait-by-field and trait-by-unit residual effects, a diagonal structure was used to account for trait-specific variances while assuming no covariance between traits. These two terms were simplified due to convergence issues and data sparsity, which precluded fitting a full unstructured variance–covariance structure. This approach allows estimation of trait-specific genetic variances and covariances from the trait-by-clone effect, from which genetic correlations ${r}_{\text{g}}$ between traits were derived using the standard formula:

3 $${r}_{\text{g}}=\frac{{\text{Cov}}_{\text{clone}}(T1,T2)}{\sqrt{{\text{Var}}_{\text{clone}}\left(T1\right) . {\text{Var}}_{\text{clone}}(T2)}}$$

where ${\text{Cov}}_{\text{clone}}(T1,T2)$ is the estimated genetic covariance between traits *T*1 and *T*2, and ${\text{Var}}_{\text{clone}}\left(T\right)$ is the genetic variance for trait *T*.

## Results

### Phenotypic variation across years and varietal clonal populations

**Fig. 1:**
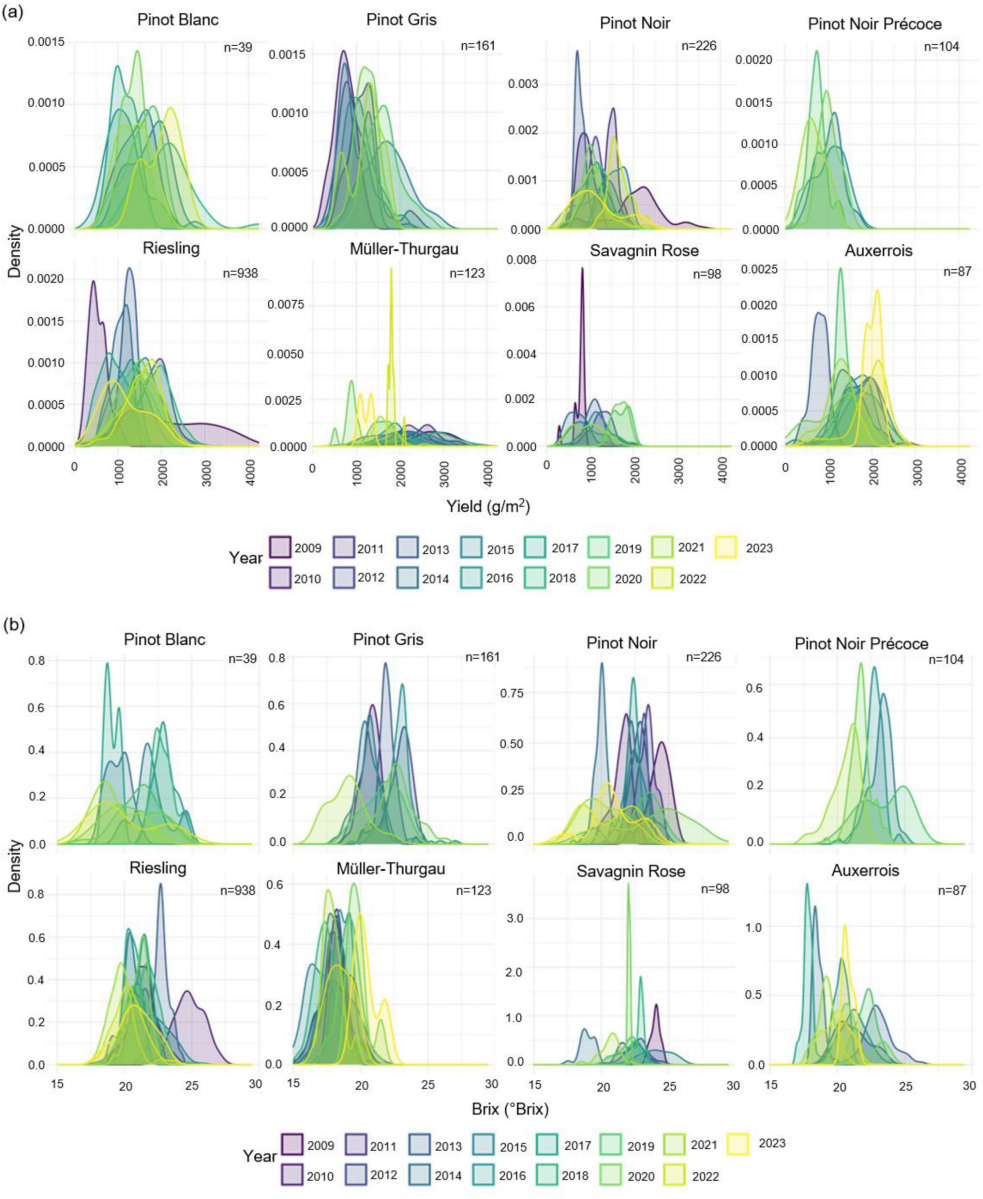
Year-specific distributions of **a** yield and **b** brix for each varietal population, Pinot Blanc (*n* = 39), Pinot Gris (*n* = 161), Pinot Noir (*n* = 226), Pinot Noir Précoce (*n* = 104), Riesling (*n* = 938), Müller-Thurgau (*n* = 123), Savagnin Rose (*n* = 98) and Auxerrois (*n* = 87), where *n* is the number of unique clones per variety. Colours represent different years from earliest (purple/blue) to most recent (yellow/green), illustrating both year-to-year variation and distinct population-specific patterns

When raw trait observations were separated based on year, substantial phenotypic variation was observed for all traits, both within individual years and across all years measured in each varietal clonal population (Fig. 1; Supplementary Fig. 3). Figure 1 shows the distribution of (a) Yield and (b) Brix values across years for each varietal population, highlighting both year-to-year variation and distinct population-specific distribution patterns. For brix some populations, such as Pinot Gris, Pinot Noir and Müller-Thurgau, maintain relatively stable central values across years, whereas in Pinot Blanc and Riesling both the mean and spread vary noticeably over time. Skewness and distribution shape also differ among populations, with Auxerrois and Savagnin Rose showing more pronounced skew across yield and brix in some years, while Müller-Thurgau displays comparatively symmetric distributions for yield. For brix consistent temporal trends are apparent in several populations, with more recent years (yellow and light green) in Pinot Blanc, Pinot Gris, Pinot Noir, and Riesling exhibiting broader, flatter distributions, whereas earlier years tend to display sharper, more pronounced peaks. Similar patterns were observed for the remaining traits (Supplementary Fig. 3A–D).

### Clonal effect contributes to phenotypic variation for complex traits

**Fig. 2:**
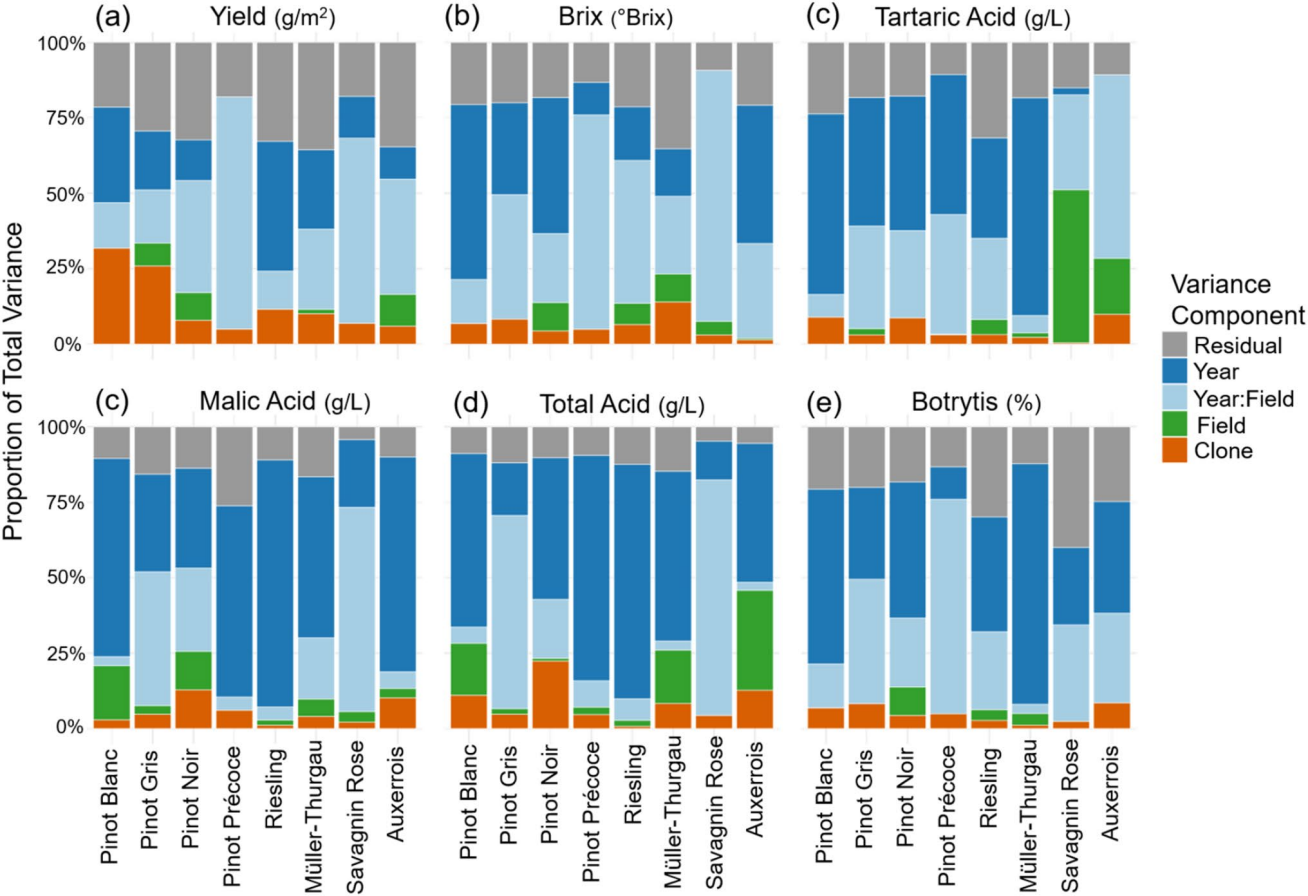
Proportional contribution of variance components to total phenotypic variance for six traits across all clonal populations. Each bar represents a varietal clonal population, Pinot Blanc (*n* = 39), Pinot Gris (*n* = 161), Pinot Noir (*n* = 226), Pinot Noir Précoce (*n* = 104), Riesling (*n* = 938), Müller-Thurgau (*n* = 123), Savagnin Rose (*n* = 98) and Auxerrois (*n* = 87), where *n* is the unique number of clones per variety, and variance components are shown as percentage contributions to total variance (full bar) and include clonal (orange), field (green), the average year-by-field interaction (light blue), year (dark blue) and the average residual at the year-by-plot-level (grey) effects. Each full bar represents 100% of the total variance for the random model effects for **a** yield, **b** brix, **c** tartaric acid, **d** malic acid, **e** total acid and **f** botrytis bunch rot

Variance parameters were estimated from the multi-year linear mixed model fit individually for each trait within each clonal population, and the proportional breakdown of these specific sources of variation are presented in Fig. 2. Across populations, the contribution of clonal effect to total variance was generally largest for yield, followed by brix and total acid content, while botrytis susceptibility had the smallest effect. Within a trait, variance in the clonal contribution is evident, for instance, in the Pinot Blanc and Pinot Gris populations the clonal contribution to yield is 32.6% and 25.1%, respectively, while lower at 13.5% and 6.0% in Riesling and Auxerrois (Supplementary Table 3). Similarly, for brix, the clonal contribution was highest in Müller-Thurgau at 14.0%, followed by Pinot Gris at 8.4%, while Auxerrois had the lowest clonal contribution at 4.3%. The proportion of variance attributed to the year and year-by-field effects are substantial and predominate for most traits, where the year effect is dominate for malic (ranging from 22.5 to 81.8%) and total acid content (12.8 to 77.7%) for most populations. On its own, the contribution of field has a small effect on total variance with the exception of tartaric acid, and in particular for the Savagnin Rose population with 51.0% of the variance explained by field term. The residual variance ranged from 4.3 to 80.1% across traits and populations, while the largest proportion of residual variance was observed for botrytis susceptibility in Pinot Précoce, followed by yield for Pinot Noir at 42.5%.

**Fig. 3:**
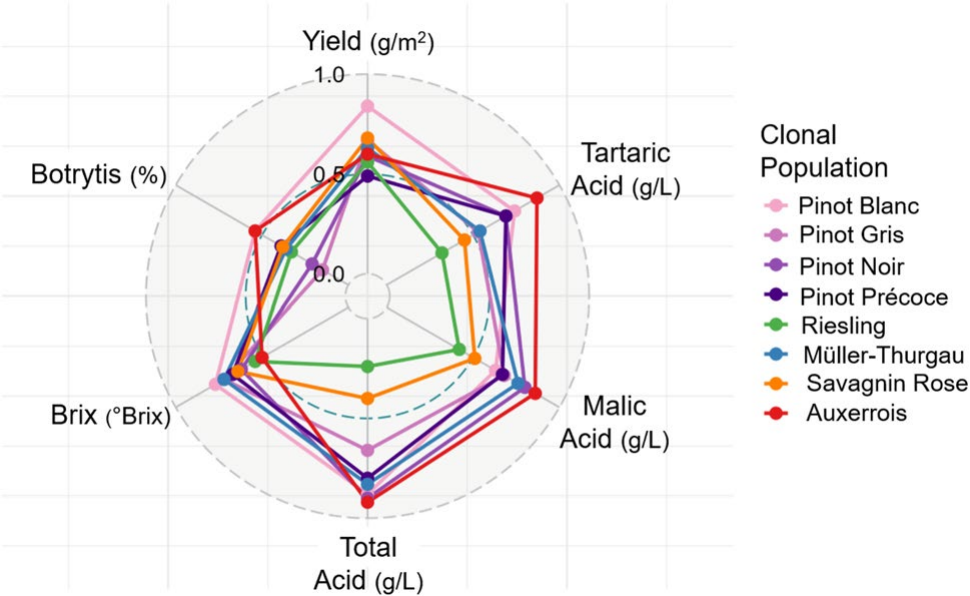
Radar graph of broad-sense heritability (H^2^) for six traits across all clonal populations, where H^2^ of each trait for a variety is connected with a coloured line for the Pinot Blanc (*n* = 39), Pinot Gris (*n* = 161), Pinot Noir (*n* = 226), Pinot Noir Précoce (*n* = 104), Riesling (*n* = 938), Müller-Thurgau (*n* = 123), Savagnin Rose (*n* = 98) and Auxerrois (*n* = 87), where *n* is the unique number of clones per variety. The heritability value increase from the inner to outer circle, from 0.00 to 1.00, with 0.50 represented by a thin blue dotted line

Given the unbalanced nature of the multi-year dataset, variance component estimates for the clonal effect must be interpreted considering both cumulative replication across years and the pairwise prediction error variance, as the latter directly impacts the reliability of ranking clones for accurate selection. To account for this, broad-sense heritability was calculated using the method described by Cullis et al. (2006). Heritability estimates across populations ranged from 0.47 to 0.84 for yield, 0.50 to 0.77 for brix, 0.13 to 0.87 for tartaric Acid, 0.24 to 0.92 for total acid, 0.42 to 0.86 for malic acid and 0.15 to 0.54 for botrytis susceptibility (Table 1; Fig. 3). While re-ranking for trait-specific heritabilities occurred across populations, trends remained relatively consistent for seemingly related traits, such as tartaric acid, malic acid, and total acid content (Fig. 3). Yield showed comparatively high genotypic coefficients of variation (CV_G_%) across several populations, particularly Pinot Blanc (18.4%) and Pinot Gris (18.5%), indicating substantial clonal differentiation in this trait (Table 1). Notably, Pinot Noir exhibited elevated CV_G_% for the acid traits, with 11.2% for total acidity and 14.7% for malic acid, suggesting marked intra-varietal diversity in acid profiles within this population.

**Table 1:** Trait summary statistics at variety level, including mean E-BLUPs, genotypic coefficient of variation (CV_G_%) and broad-sense heritability (*H*^2^)

Trait	Variety	Mean	CV_G_%	*H*^2^
Yield (g/m^2^)	Pinot Blanc	1,574.81	18.40	0.84
	Pinot Gris	1,151.59	18.51	0.67
	Pinot Noir	1,100.21	10.40	0.59
	Pinot Noir Précoce	1,012.26	14.87	0.49
	Riesling	1,387.71	13.48	0.56
	Müller-Thurgau	2,111.16	9.15	0.63
	Savagnin Rose	975.11	12.22	0.47
	Auxerrois	1,714.82	8.13	0.60
Brix (°Brix)	Pinot Blanc	20.72	2.46	0.77
	Pinot Gris	21.76	2.41	0.72
	Pinot Noir	21.88	2.01	0.62
	Pinot Noir Précoce	22.26	1.89	0.67
	Riesling	21.48	2.03	0.54
	Müller-Thurgau	18.17	2.57	0.72
	Savagnin Rose	22.53	1.29	0.52
	Auxerrois	20.16	1.03	0.50
Total acid (g/L)	Pinot Blanc	7.85	7.88	0.86
	Pinot Gris	8.75	4.69	0.66
	Pinot Noir	7.69	11.24	0.90
	Pinot Noir Précoce	7.47	4.89	0.80
	Riesling	10.05	2.12	0.24
	Müller-Thurgau	7.41	4.89	0.83
	Savagnin Rose	7.25	6.42	0.73
	Auxerrois	7.60	6.48	0.92
Tartaric acid (g/L)	Pinot Blanc	5.41	6.35	0.74
	Pinot Gris	5.57	4.03	0.53
	Pinot Noir	4.72	7.66	0.69
	Pinot Noir Précoce	4.92	6.71	0.69
	Riesling	6.31	3.62	0.32
	Müller-Thurgau	5.30	3.11	0.54
	Savagnin Rose	4.96	1.45	0.13
	Auxerrois	6.03	4.34	0.87
Malic acid (g/L)	Pinot Blanc	2.83	10.64	0.63
	Pinot Gris	3.85	8.86	0.68
	Pinot Noir	3.81	14.66	0.80
	Pinot Noir Précoce	3.08	7.96	0.67
	Riesling	4.20	5.12	0.42
	Müller-Thurgau	2.81	6.88	0.76
	Savagnin Rose	3.15	6.70	0.64
	Auxerrois	2.33	12.60	0.86
Scaled Botrytis (%)	Pinot Blanc	1.15	3.64	0.54
	Pinot Gris	0.74	1.90	0.15
	Pinot Noir	0.74	2.56	0.21
	Pinot Noir Précoce	0.69	0.86	0.39
	Riesling	2.29	1.33	0.33
	Müller-Thurgau	1.11	5.65	0.36
	Savagnin Rose	1.40	7.49	0.52
	Auxerrois	1.50	8.38	0.54

### Wide phenotypic variance persists across traits in adjusted clonal estimates

**Fig. 4:**
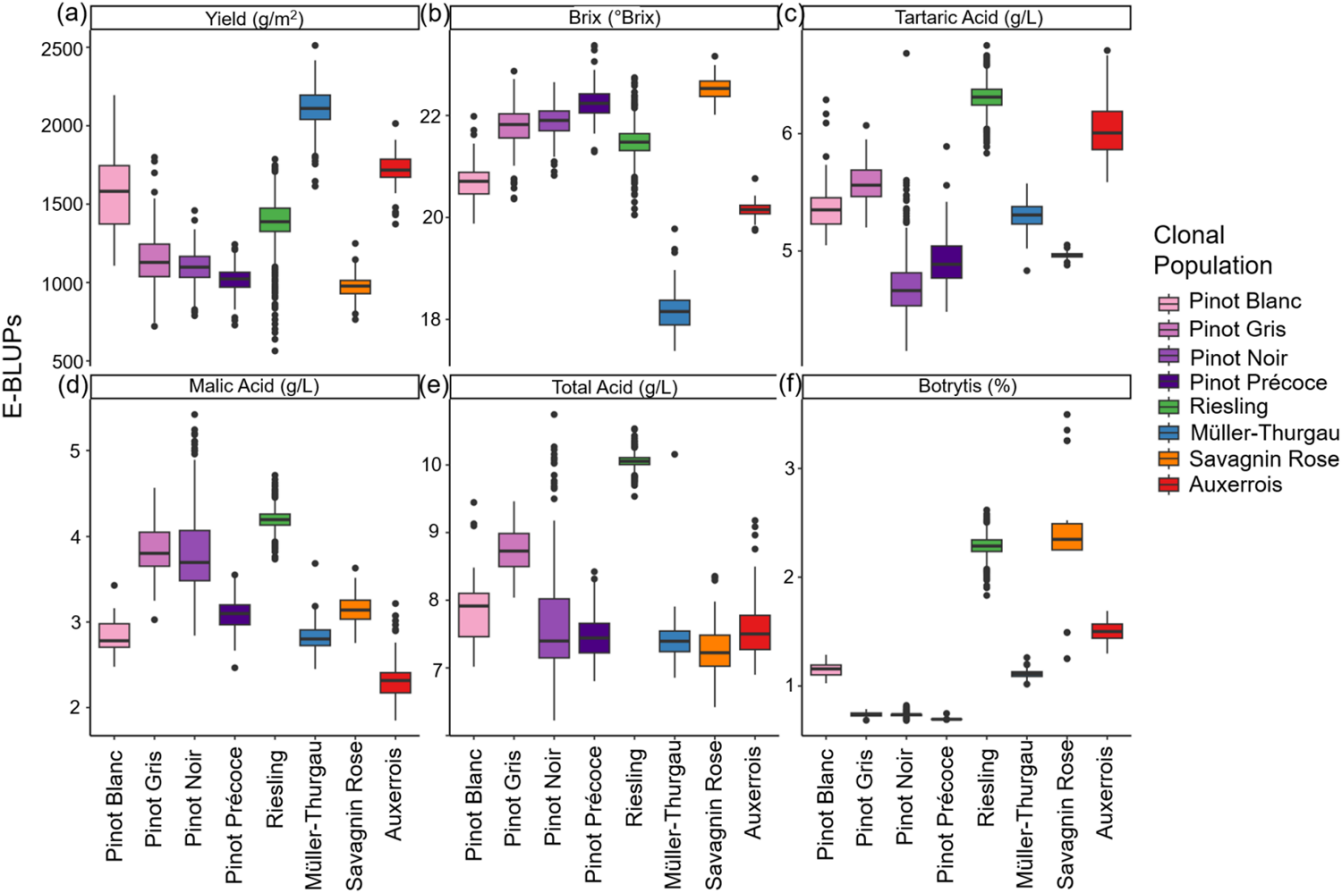
Boxplots of empirical best linear unbiased predictors (E-BLUPs) of clonal genetic effects from the multi-year linear mixed model, adjusted by adding the model intercept to represent variety means for each trait: **a** yield (g/m^2^), **b** brix (°Brix), **c** tartaric acid (g/L), **d** malic acid (g/L), **e** total acid (g/L), and **f** scaled botrytis bunch rot (%). Varieties shown are Pinot Blanc (*n* = 39, *y* = 9), Pinot Gris (*n* = 161, *y* = 9), Pinot Noir (*n* = 226, *y* = 14), Pinot Noir Précoce (*n* = 104, *y* = 5), Riesling (*n* = 938, *y* = 14), Müller-Thurgau (*n* = 123, *y* = 12), Savagnin Rose (*n* = 98, *y* = 9) and Auxerrois (*n* = 87, *y* = 9), where *n* is the unique number of clones per variety and *y* is the unique number of years of data. As E-BLUPs were predicted separately for each variety, formal statistical comparisons between varieties are not appropriate

The multi-year mixed model analysis revealed considerable variance in the predicted E-BLUPs across all traits for all clonal populations (Fig. 4). Trait-specific population performance exhibited clear re-ranking patterns, for example, Auxerrois and Riesling populations had the highest tartaric acid content E-BLUPs (Fig. 4c), while Auxerrois and Müller-Thurgau populations showed the lowest sugar content (Fig. 4b) and highest yield (Fig. 4a). Similar to the population heritability trends, rankings remained reasonably consistent across acid content traits, whereas an inverse relationship was observed between yield and brix (Fig. 4a and b). Variance in predictions across traits are observed within the Pinot varieties, particularly for yield and the acid content traits. For Botrytis bunch rot, greater within-population variance was evident in Riesling, Savagnin Rose and Auxerrois compared to the narrower ranges observed in the Pinot varieties and Müller-Thurgau. Given the trait-by-trait, population-specific nature of the analysis, statistical significance tests between population E-BLUPs were not applicable.

### Negative correlation between yield and quality traits predominates across clones

**Fig. 5:**
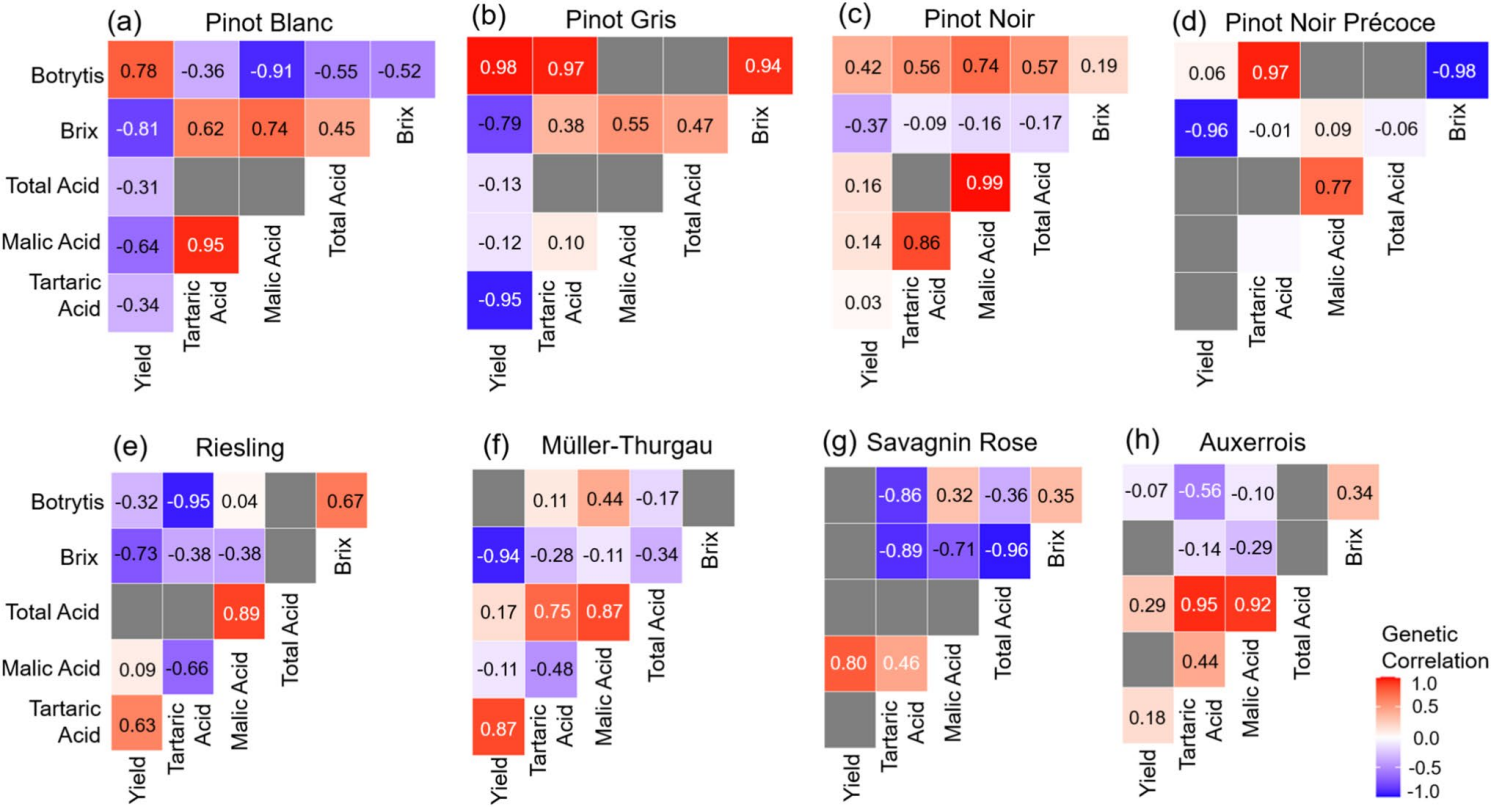
Heatmaps of pairwise genetic correlations between traits within each varietal clonal population: **a** Pinot Blanc (*n* = 39), **b** Pinot Gris (*n* = 161), **c** Pinot Noir (*n* = 226), **d** Pinot Noir Précoce (*n* = 104), **e** Riesling (*n* = 938), **f** Müller-Thurgau (*n* = 123), **g** Savagnin Rose (*n* = 98) and **h** Auxerrois (*n* = 87), where *n* is the unique number of clones per variety. Correlation strength and direction are colour-scaled from strong negative (− 1; blue) to strong positive (1; red), with zero correlation represented in white. Grey tiles indicate trait combinations where genetic correlations could not be reliably estimated

A number of genetic correlations were consistent across clonal populations (Fig. 5), most notably the strong negative relationship between yield and brix, which ranged from − 0.37 to − 0.96. The relationship between acid content traits (tartaric acid, malic acid, and total acidity) and yield was generally positive, particularly for tartaric acid in Riesling and Müller-Thurgau populations, but was negative in Pinot Blanc and Pinot Gris. Acid content traits were predominately positively correlated, except in Riesling and Müller-Thurgau, where negative correlation (− 0.66, and − 0.48, respectively) were detected between malic and tartaric acid. A consistent negative relationship between brix and acid content traits was observed across all populations, with the exception of Pinot Blanc and Pinot Gris, where moderate positive correlations were detected, and Pinot Précoce, where correlations were weak. Genetic correlations were estimated using the full clonal populations reported in Supplementary Table 1 (ranging from 39 clones in Pinot Blanc to 938 clones in Riesling). While several strong and consistent correlations were detected across populations, others could not be reliably estimated due to data imbalance and low genetic variance, and relationships involving Botrytis were particularly variable across traits and populations, reflecting the lower reliability of estimates for this trait.

### E-BLUPs reveal potential for within-population clonal selection

**Fig. 6:**
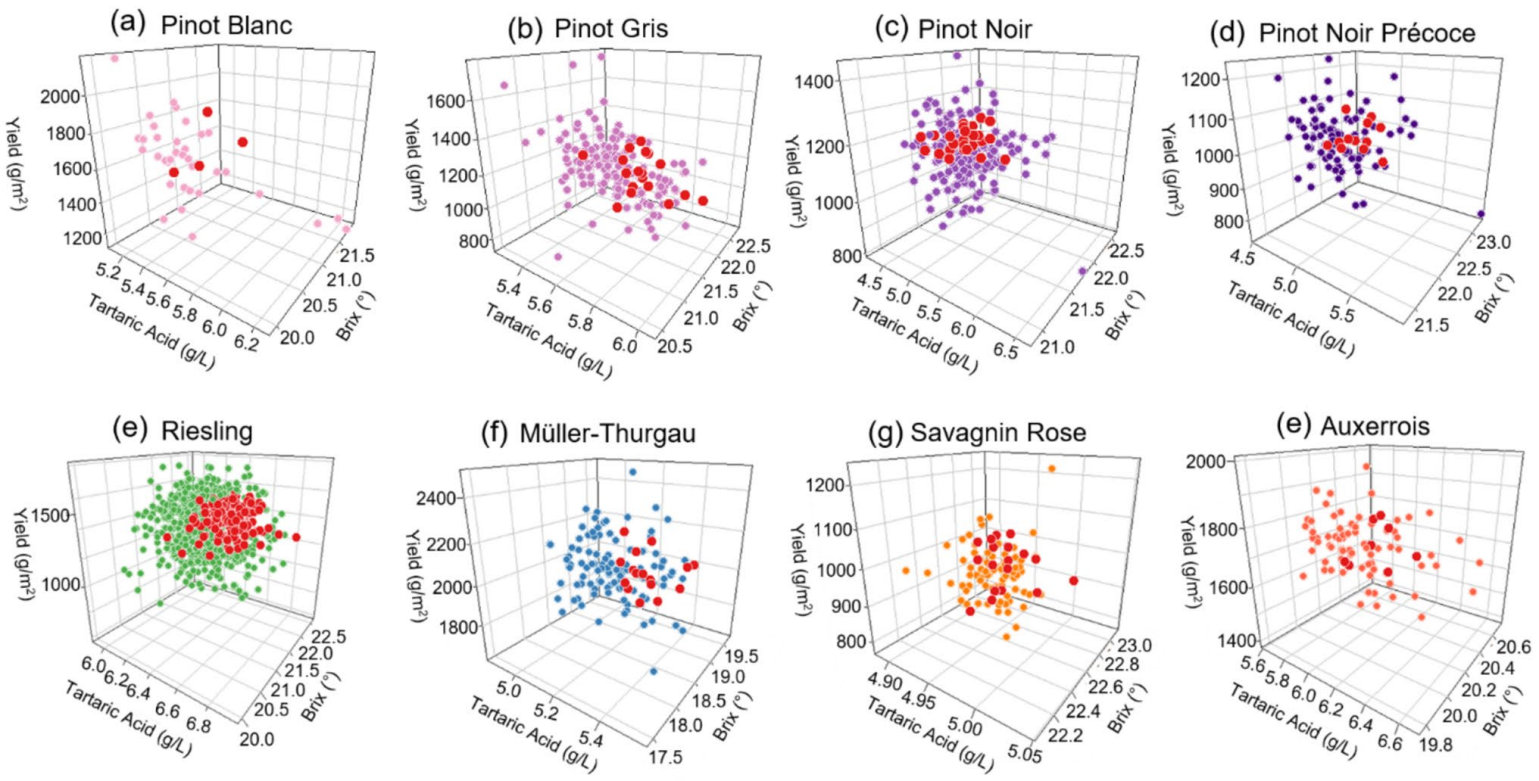
Three-dimensional bi-plots displaying multi-year E-BLUPs adjusted by adding the model intercept to represent variety means for yield (z-axis), tartaric acid (*x*-axis), and brix (*y*-axis) across eight varietal clonal populations: **a** Pinot Blanc, **b** Pinot Gris, **c** Pinot Noir, **d** Pinot Précoce, **e** Riesling, **f** Müller-Thurgau, **g** Savagnin Rose, **h** Auxerrois. Clones highlighted in red meet the theoretical selection criteria: top 30th percentile for tartaric acid, bottom 60th percentile for brix, and within the 30th to 95th percentile for yield. A total of 4 Pinot Blanc, 19 Pinot Gris, 29 Pinot Noir, 12 Pinot Précoce, 132 Riesling, 69 Pinot, 19 Savagnin Rose, 16 Müller-Thurgau, and 8 Auxerrois clones met these selection thresholds

Visualisation of within-population E-BLUPs for tartaric acid, brix, and yield highlights the potential for targeted clonal selection within each varietal population (Fig. 6). Using arbitrary selection thresholds (top 30% for high tartaric acid, bottom 60% for Brix, and the 30–95% range for yield) potential selections were identified across all populations. This could help to identify new clones with lower sugar, higher tartaric acid and good yield potential that could be more suitable for constantly warming climates in the future. The proportion of clones meeting these criteria varied across populations (Supplementary Table 4), ranging from 10.3% in Pinot Blanc, 11.8% in Pinot Gris, 12.8% in Pinot Noir, and 11.5% in Pinot Précoce, to 14.1% in Riesling, 19.4% in Savagnin Rose, 13.0% in Müller-Thurgau, and 9.2% in Auxerrois. While this selection approach is a preliminary assessment, it demonstrates the potential for within-population clonal selection to enhance key agronomic and quality traits.

## Discussion

This study provides a comprehensive, long-term assessment of intra-varietal phenotypic variation by examining six key traits within eight commercially important clonal populations over more than a decade. By quantifying the proportion of variance attributable to the clone effect, our analysis provides unique insights into the potential for genetic improvement via clonal selection across these complex traits. Substantial phenotypic variation was evident across all populations, with meaningful clonal variance components detected for most traits (Table 1, Supplementary Table 3), confirming that intra-varietal diversity is a consistent feature across both widely cultivated and regionally important cultivars.

### Contextualising findings within existing literature

To our knowledge, several studies have examined phenotypic variation within clonal populations of grapevine, including in Arinto (Carvalho et al. 2025), Cabernet Franc (Van Leeuwen et al. 2013), Grenache (Buesa et al. 2021), Malbec (van Houten et al. 2020), Tempranillo (Arrizabalaga et al. 2018; Portu et al. 2024),White Riesling (Laidig et al. 2009) and ancient grapevine varieties (Gonçalves and Martins 2022). Most of these studies assessed traits such as yield, brix, and total acidity, providing a useful basis for comparison. While direct comparisons are complicated by differences in experimental design and data reporting, general trends suggest that our study exhibits comparatively high phenotypic variance for traits like yield and total acidity. This may be attributable to the larger population sizes included here, the presence of older cultivars, and generally the broader temporal coverage of phenotypic data, which spans a period of increasing climatic variability. Overall, these comparisons support the conclusion that the eight clonal populations analysed in this study capture substantial phenotypic diversity for key breeding traits.

A key contribution of this study is the quantification of substantial genetic variance attributable to clonal differences, with broad-sense heritability (H^2^) frequently exceeding 0.5 across the studied clonal populations. While previous grapevine clonal population studies often described phenotypic variation, several fail to partition the underlying variance components to explore the contribution of individual effects, particularly the clonal genetic component. Laidig et al. (2009), however, provided valuable estimates for Riesling, finding small but statistically significant clonal variance components (less than 1% for yield and quality traits) among 30 commercial clones, suggesting limited genetic differentiation. In contrast, our results demonstrate considerably larger clonal effects, especially within the expansive Riesling population analysed here (938 clones), where clonal variance accounted for 14.55% (yield) and 6.50% (brix) of phenotypic variance (Supplementary Table 3). This discrepancy likely reflects the broader genetic and phenotypic spectrum captured in our study, which included both widely cultivated and historically underutilised clones, many of which were identified during targeted surveys of heritage vineyards. Whereas Laidig et al. (2009) focused on commercial clones already subject to intensive clonal selection for target traits, our collection encompasses a more diverse representation of the clonal landscape. We acknowledge that the partial replication of clones across years may inflate broad-sense heritability estimates; however, this effect could be counterbalanced by the extensive temporal coverage of our dataset, which provides a good basis for robust inference.

The study also highlighted the contribution of other key variance components across the multi-year analysis, and most notably the impact of year and the year-by-field interaction for all traits. This result is not unsurprising, with several prior studies in grapevine reporting the significant impact of year-to-year variation on agronomic, phenology and quality-related traits (Costantini et al. 2008; Laidig et al. 2009; Suter et al. 2021). The high year-to-year variance observed for malic acid (ranging from 22.5 to 82.9% of total phenotypic variance; Fig. 3) is likely due to its metabolic instability and sensitivity to environmental conditions, with warmer seasons accelerating degradation and cooler temperatures slowing the process, resulting in substantial annual variation (Rienth et al. 2016). Gonçalves et al. (2016) demonstrated that genotype-by-environment interactions are significant in clonal populations across traits such as yield, probable alcohol, and acidity content, emphasising its critical role in clonal selection pipelines. Notably, Gonçalves et al. (2016) also found the year effect to be substantial, with genotype performance across different years at the same site being no more correlated than across different sites, highlighting the importance of accounting for temporal variation. Consistent with this emphasis on temporal influence, our results similarly demonstrated that the combined variance components for year and year-by-location interaction substantially exceeded that of the field effect alone across the studied traits and populations.

Our analysis of genetic correlations, while acknowledging the inherent estimation challenges associated with complex, unbalanced clonal datasets (discussed below), reveals both biologically meaningful patterns and points of divergence from previous grapevine studies. Consistent negative genetic correlations were observed between yield and brix, across all populations (e.g. Riesling − 0.73; Pinot Blanc − 0.81; Pinot Gris − 0.79), supporting previously reported tendencies towards a trade-off between productivity and sugar accumulation (Damiano et al. 2022). Recent polyclonal selection work by Surgy et al. (2025) similarly reported strong negative phenotypic correlations between sugar concentration and total acidity, a relationship that is also evident at the genetic level in our study, where brix was negatively correlated with both malic and tartaric acid content across most populations, except Pinot Blanc and Pinot Gris. This convergence of phenotypic and genetic signals across different populations underscores the biological significance of the sugar–acid balance in grapevine berry development and ripening.

Interestingly, we observed a strong negative genetic correlation between tartaric and malic acid content in Riesling (− 0.66) and Müller-Thurgau (− 0.48), which contrasts with expectations given the acid’s largely independent biosynthetic pathways. As malic acid degradation is highly sensitive to temperature and other environmental factors (Sweetman et al. 2014), this unexpected relationship may reflect population-specific co-selection or environmental co-variation rather than true metabolic antagonism. Additional targeted studies under controlled conditions would be necessary to disentangle these effects. Also noteworthy is the consistently strong positive genetic correlation between tartaric acid and total acidity, suggesting tartaric acid is the dominant driver of total acidity under the sampling conditions used here. In contrast, the contribution of malic acid to total acidity appears more variable across populations. Finally, the Botrytis bunch rot showed a strong negative genetic correlation with brix in some populations (e.g. Savagnin Rose − 0.89; Riesling − 0.95), which may reflect both physiological and sampling-based artefacts. As botrytis scoring may have varied across years and was likely confounded by harvest timing (discussed below), caution is warranted in interpreting these relationships as causal.

Multi-year E-BLUPs revealed that all clonal populations generally exhibited higher levels of tartaric acid relative to malic acid, except for some Pinot Noir clones (Fig. 4), where malic and tartaric acid were present at more comparable levels. This intra-population variation suggests that certain Pinot Noir clones possess greater malic acid stability. These findings point to opportunities for selecting clones with more favourable acid balance, a key trait for climate-adaptive breeding. Furthermore, improving our understanding of the genetic relationships between malic and tartaric acid content will enhance selection precision, enabling breeders to better predict acid profiles and optimise clonal performance across both cool and warm growing regions.

### Breeding implications and selection strategies

The substantial phenotypic variance directly attributable to the genetic clonal effect across the studied populations confirms the feasibility of achieving genetic gains for complex traits via clonal selection. This finding holds particular significance for the wine industry, which often exhibits conservatism towards adopting entirely new cultivars yet faces pressing demands to adapt established, high-value varieties to evolving climate challenges. Utilising the inherent diversity within existing clonal populations, as demonstrated here, offers an important pathway for enhancing adaptation and performance while maintaining variety integrity. Furthermore, identifying traits with low clonal variance within these populations is equally valuable, informing targeted breeding strategies, whether exploiting natural mutations or employing advanced breeding techniques, to introduce necessary genetic diversity where it is currently lacking for future improvement goals.

Effectively harnessing the identified clonal variation for tangible genetic improvement, particularly for complex goals like climate adaptation, necessitates selecting across multiple traits simultaneously. Targeted selection for acid and sugar balance for future climate adaptation is a key example of the multi-trait selection opportunities that are apparent within the diversity present in these populations. Using a basic example of three traits with simplistic selection criteria we have demonstrated the ability to select high performing clones in all eight clonal populations. While the exact quality trait configurations for adaptation of major varieties like Riesling to future environments requires further exploration, it is apparent that it will be highly complex and multifaceted, and thus may necessitate the use of selection indices, similar to the multi-trait genotype-ideotype distance index approached applied by Brault et al. (2024) in Rosé wine and Cognac production breeding programs.

Although the aim of selection indices is to identify the best clones based on multiple traits, a key limitation lies in the risk of improving certain traits at the expense of others. This risk is particularly pronounced, and can increase substantially, when negative genetic correlations exist among traits, a reality that is explicitly evident in the current study. As an alternative, Surgy et al. (2025) propose the use of optimisation frameworks that use polyclonal selections rather than single clone selections, specifically using integer programming, to enable effective multi-trait selection in breeding scenarios. By allowing the definition of realistic minimum gains, this approach facilitates the selection of groups of clones that achieve improvements in desirable traits while avoiding losses in others. The extension of such optimisation-based approaches to multi-trait clonal selection offers considerable potential, especially for heritage grapevine varieties where centuries of vegetative propagation have generated broad clonal diversity that can be strategically harnessed through polyclonal selection.

## Limitations and future directions

A key limitation of this study is the lack of a replicated experimental design in the historical dataset, particularly the absence of within-field replication and the minimal within-year replication, that is further compounded by inconsistent sampling across years (Supplementary Table 1). While this limitation is somewhat mitigated by the extensive dataset, with all but one clonal population having at least 9 years of trait data and some up to 14 years, a more structured and ideally fully replicated experimental design would improve the precision of variance estimates, particularly in perennial species where environmental deviations may be high. Implementing even a partially replicated (p-rep) design, as demonstrated by Gonçalves et al. (2022), or a multi-environment augmented p-rep design (Moehring et al. 2014), could reduce prediction error variance and enhance the efficiency of clonal selection, particularly in early-generation evaluations. Furthermore, when combined with genomic selection approaches that leverage genomic relationship matrices, partial replication across genotypes and years can likely provide effective training populations in perennial clonal crops.

Despite the lack of formal replication, the dataset does contain multiple years of repeated observations for all clones, and partial replication across plots within years. This extensive temporal coverage provides an important source of replication that strengthens the estimation of clonal effects over time, even where within-year replication is limited. The inclusion of multiple fields also broadens the environmental basis for inference, although the resulting imbalance and sparsity remain important considerations when interpreting estimates of variance components and heritability. Looking ahead, the integration of genomic information could further enhance the reliability of these estimates by helping to disentangle genetic from environmental contributions, particularly in unbalanced or partially replicated datasets.

Rootstock use is another potential source of confounding in clonal evaluations, as scion–rootstock interactions are known to influence phenotypic expression. In our dataset, rootstock identity was either completely confounded with field (i.e. one rootstock per field) or largely dominated by a single rootstock type (Supplementary Table 1). As a result, rootstock could not be fitted as an independent factor in the mixed models. Instead, the field effect, modelled as a random factor, absorbs the majority of this confounding. While this approach does not allow the separation of rootstock-specific contributions from broader field effects, it does ensure that variation attributable to rootstock differences is not conflated with the clone effect.

In addition to the limitations of replication and rootstock confounding, the sparse and spatially dispersed arrangement of plots limited our ability to effectively model local environmental heterogeneity. Consequently, uncaptured spatial effects likely inflated residual variances and uncertainty in the genetic estimates. This data structure not only increases uncertainty and shrinkage in predictions but can also compromise genetic correlation estimates derived from bivariate models. Specifically, unmodeled environmental factors co-varying between traits within plots may generate spurious covariance potentially misattributed by the model to the genetic covariance component. Such misattribution can lead to inflated estimates of genetic correlation, an effect likely exacerbated in the populations with smaller sample sizes where reliable correlation estimates were particularly challenging and not reported when reaching the boundary. Additionally, interpretation of the Botrytis bunch rot trait should be made with caution, as variation in scorer training across years and early harvest practices (especially in Auxerrois, Müller-Thurgau, and Savagnin Rose) may have limited the expression of post-veraison infection symptoms. While this necessitates caution regarding the precise magnitude of some correlations, the general significance and directional trends observed likely remain valid indicators of underlying genetic relationships.

A further analytical limitation involves the assumption of an independent diagonal variance structure for the year-by-plot residual term, which fails to explicitly model the inherent temporal dependencies expected in perennial crops like grapevines, consequently neglecting potential auto-correlation within plots across years (Gonçalves et al. 2020). Although the simplified structure adopted in our study facilitated robust model convergence across all datasets, particularly where data sparsity precluded the stable estimation of more complex covariance structures, it likely leads to an underestimation of true year-to-year correlations within plots. Such an assumption can inflate the residual variance estimate by absorbing persistent plot-level effects into the error term, potentially obscuring systematic genotype-by-year interactions and resulting in an underestimation of the temporal stability of clonal performance. Future work incorporating appropriate temporal covariance structures, where data permit, would provide more accurate insights into clonal stability over time.

Notwithstanding the discussed limitations, this study fundamentally underscores the considerable potential for achieving genetic improvement via clonal selection within established grapevine varieties, a process potentially accelerated by integrating new breeding technologies. Beyond direct selection efforts, these clonal populations represent invaluable resources for dissecting the genetic architecture of complex traits and potentially mapping causal variants with high precision. Functionally akin to near-isogenic lines, the subtle genetic and epigenetic distinctions between clones provide a powerful framework for association analyses designed to disentangle the phenotypic contributions of specific sequence or methylation changes. Insights gained from such analyses could subsequently guide the application of highly targeted precise trait improvement using genome editing tools, such as CRISPR/Cas9 that has already successfully optimised for targeted trait modification in grapevine (Li et al. 2020; Ren et al. 2016; Villette et al. 2024). Ultimately, these clonal varietal populations constitute uniquely valuable genetic material, offering pathways for advancing fundamental knowledge, however efficiently selecting improved clones remains a significant practical hurdle.

The primary constraint in traditional clonal selection is the prolonged evaluation period required to accurately calculate a clone’s genetic merit for key traits, a process that typically spans 15 or more years. The application of predictive models, such as genomic selection (GS), can substantially reduce this timeframe by using genome-wide molecular markers and trained statistical models to estimate an individual’s genetic merit at early selection stages, prior to physical trait measurement. Similarly, phenomic selection (PS) uses high-throughput phenotypic data, such as spectral or imaging-based traits, and trained statistical models to predict genetic merit. While PS has been studied in diverse grapevine varieties and half-diallel breeding populations (Brault et al. 2022), it has yet to be applied in the prediction of genetic merit within clonal populations of commercial cultivars. Given the high heterozygosity of grapevine, incorporating non-additive genetic effects into predictive models is likely essential for improving selection accuracy. Supporting this, Werner et al. (2023) emphasised the importance of including non-additive effects, such as dominance, in GS models for clonal breeding programs, particularly for optimising parental selection. Their findings indicate that when dominance effects are significant, genomic predicted cross-performance (GPCP) surpasses genomic estimated breeding values (GEBVs) in maximising genetic gain. Similar conclusions were drawn by Yadav et al. (2021) in sugarcane, and insights from both studies can be applied to clonal selection programs without sexual recombination. Since clones retain their genetic makeup, leveraging both additive and dominance effects can likely improve the accuracy of estimating clonal genetic merit, particularly at early-stages. Nevertheless, a general limitation of such population-level prediction models is their inherent difficulty in capturing the influence of extremely rare or novel mutations due to insufficient statistical power.

Larger training populations help mitigate this limitation regarding rare variants by increasing the chance they are sufficiently represented for effect estimation; simultaneously, substantial population size is a fundamental requirement for achieving high overall prediction accuracy, as GS accuracy is well-known to increase with training set size (Daetwyler et al. 2012). Aforementioned, small population sizes are a persistent challenge in grapevine, and the limitations of such are apparent in the current study. A promising solution is to connect and synchronise cross-institutional efforts by sharing phenotypic, and genotypic datasets from varietal clonal populations. By pooling data across institutes and nations, larger and more diverse training populations can be developed, improving the accuracy of predictive models for selecting superior clones, particularly for traits of moderate genetic complexity such as quality attributes and disease resistance.

Significant opportunities exist to leverage predictive breeding approaches for enhanced accuracy and efficiency of clonal selection, but their impact depends on effective integration into clonal selection programs. Yet, resource limitations make it impractical for grapevine breeders to empirically test and optimise every conceivable scenario, potentially limiting their practical benefits. Simulation-based studies provide a powerful alternative, allowing breeders to assess the effects of prediction models, like GS and PS, within selection program scenarios. Tools such as genomicSimulation (Villiers et al. 2024), AlphaSim-R (Gaynor et al. 2021), and MoPS (Pook et al. 2020) enable simulations to be parameterised with real clonal selection features, using empirical estimates of variance and heritability to extend predictions beyond what is experimentally feasible.

In conclusion, this study reveals substantial intra-varietal diversity within the examined grapevine populations, with a meaningful proportion attributable to genetic differences among clones. This genetic variation represents a valuable and largely untapped resource for accelerating genetic improvement, particularly in the context of climate adaptation. Harnessing this diversity through predictive breeding and advanced multi-trait selection strategies holds considerable promise for enhancing the resilience and performance while maintaining the varietal integrity of traditional grapevine cultivars.

## Supplementary Information

Below is the link to the electronic supplementary material.

Supplementary file1 (DOCX 2953 KB)

Supplementary file2 (XLSX 944 KB)

## Data Availability

The raw phenotypic data supporting this study are provided as supplementary material.
