# Supplementary material for: Exploring intra-varietal variation for complex traits in grapevine (Vitis vinifera L.)
Source: Theor Appl Genet. 2025 Nov 14;138(12):305. doi: 10.1007/s00122-025-05088-3 (PMC12618426; doi:10.1007/s00122-025-05088-3)
Supplement: Supplementary file 1 — Supplementary file1 (DOCX 2953 KB) [file 122_2025_5088_MOESM1_ESM.docx]

**Supplementary Material**

**Supplementary Figure 1**


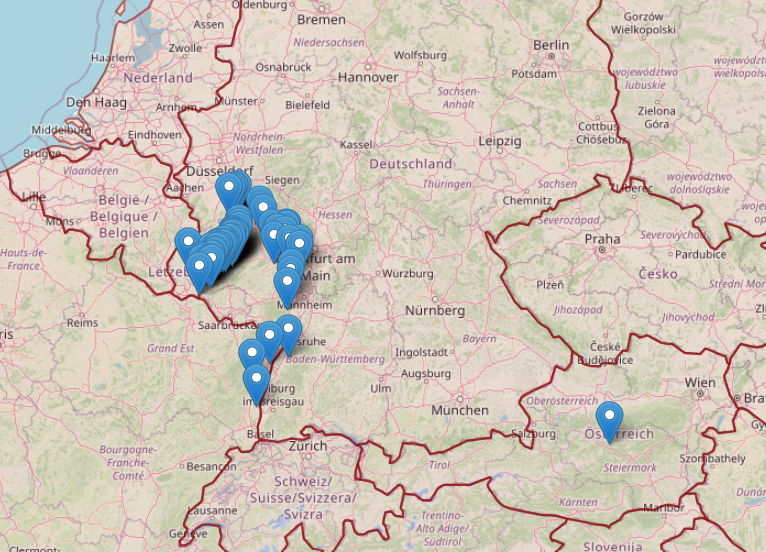


**Supp. Fig. S1** Sampling locations for the Riesling clonal population (as an example). Each blue pin marks a unique location of origin for one or several clones. The Riesling clones originate predominately from Germany but also Austria, France and Luxembourg.

**Supplementary Figure 2**

**
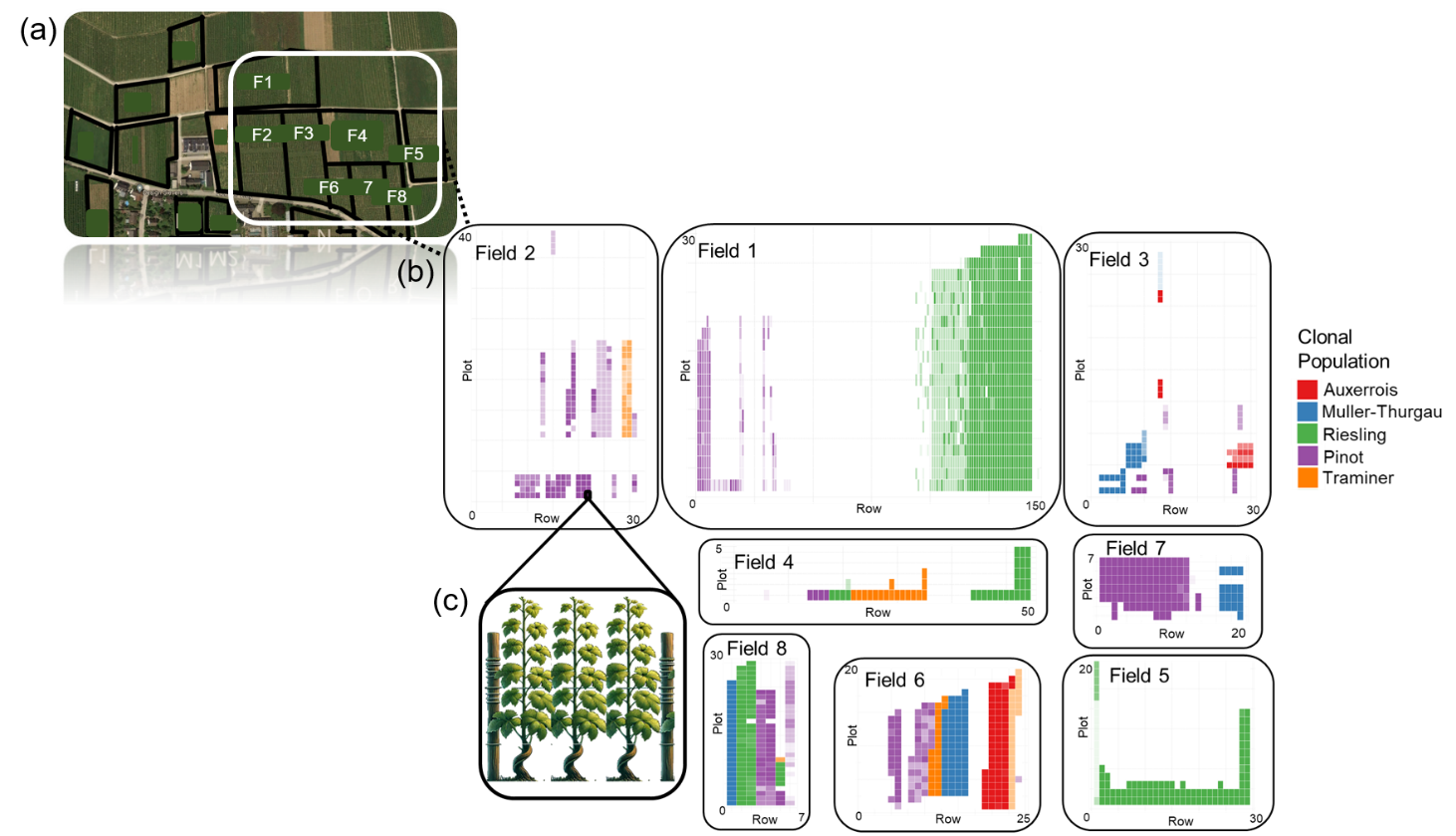
**

**Supp. Fig. S2 Overview of the experimental site and sampling structure. (a)** Aerial photograph indicating the location and orientation of the experimental fields. **(b)** Detailed plot maps for representative fields, illustrating the distribution of clonal populations (indicated by colour). The density of the plot colour reflects sampling frequency, with lighter colours representing plots sampled less often. **(c)** Schematic showing a single experimental plot containing three vines.

**Supplementary Figure 3**

**S3A**

**
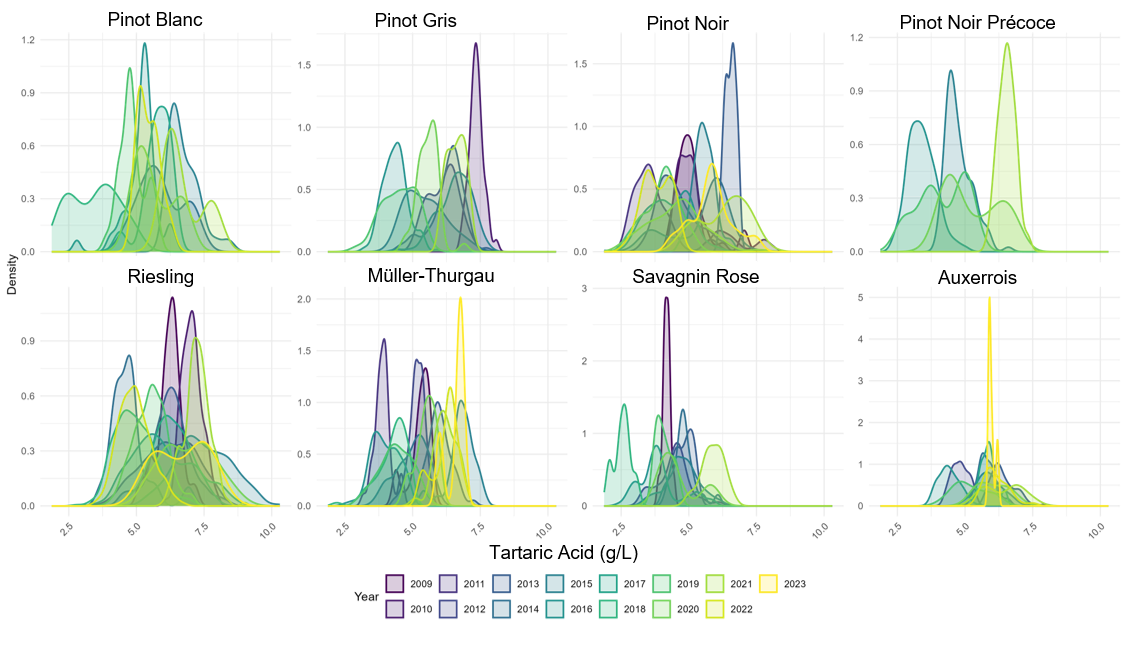
**

**S3B**

**
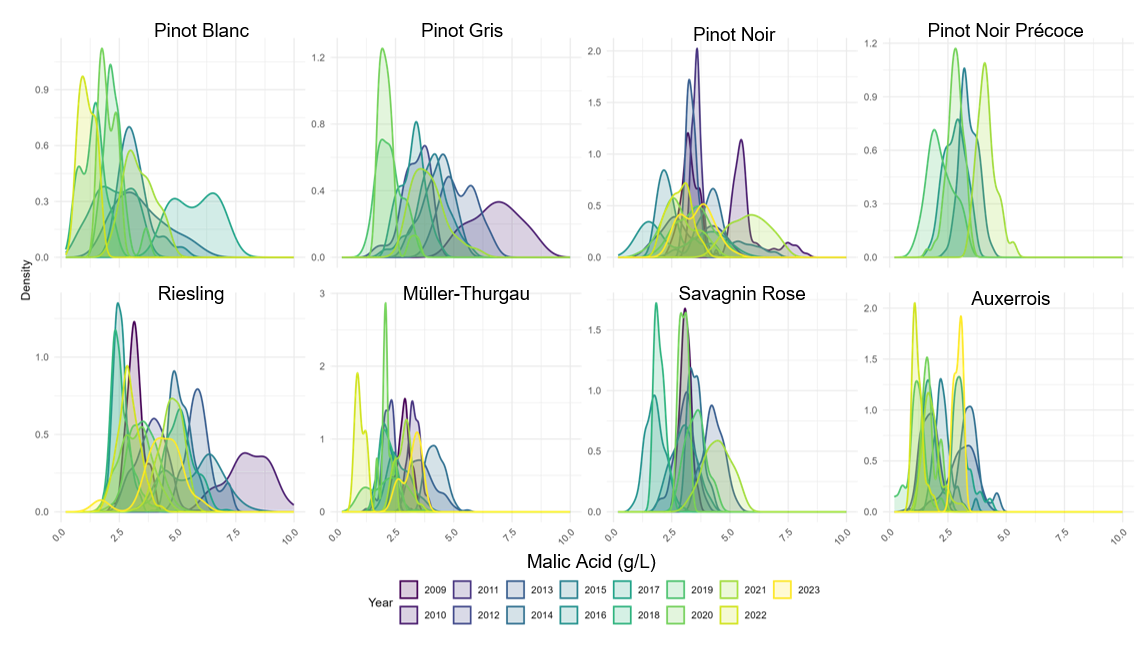
**

**S3C**

**
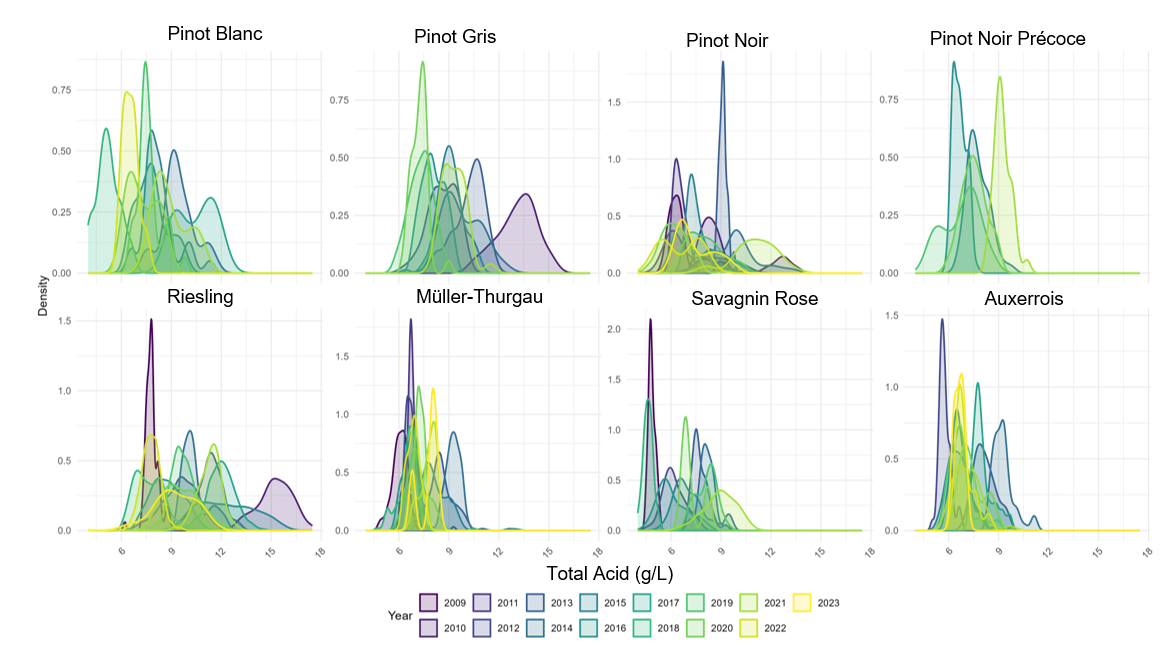
**

**S3D**

**
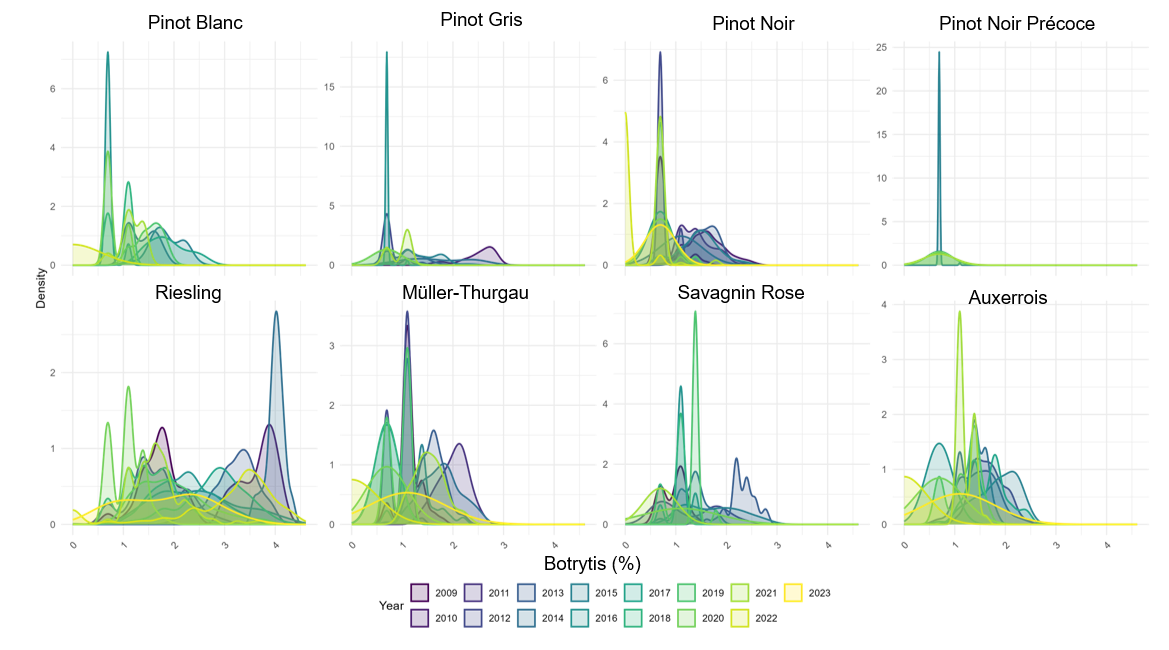
**

**Supp. Fig. S3 (A–E).** **Year-specific distributions of trait values across all varieties.** Panels correspond to traits: (A) Tartaric Acid (g/L), (B) Malic acid (g/L), (C) Total acid (g/L), and (D) transformed Botrytis susceptibility (%) for each varietal population, Pinot Blanc (n=39), Pinot Gris (n=161), Pinot Noir (n=226), Pinot Noir Précoce (n=104), Riesling (n=938), Müller-Thurgau (n=123), Savagnin Rose (n=98) and Auxerrois (n=87), where n is the number of unique clones per variety. Colours represent different years from earliest (purple/blue) to most recent (yellow/green), highlighting year-to-year variation and trait-specific patterns across the varietal populations.

**Supp. Mat. S4. Final model fit ASReml-r script**

asreml(*Trait* ~ 1,

random= ~CloneID + Year + Field + diag(Year):(Field),

residual= ~diag(Year):PlotID,

data= data.df, na.action = na.method(x = "include"))
